# Design of V_2_O_5_ Blocks Decorated with Garlic Peel Biochar Nanoparticles: A Sustainable Catalyst for the Degradation of Methyl Orange and Its Antioxidant Activity

**DOI:** 10.3390/ma16175800

**Published:** 2023-08-24

**Authors:** Perumal Sarojini, Karuppasamy Leeladevi, Thavuduraj Kavitha, Krishnamoorthy Gurushankar, Ganesan Sriram, Tae Hwan Oh, Karthik Kannan

**Affiliations:** 1 Department of Chemistry, Sri S. Ramasamy Naidu Memorial College, Sattur 626203, Tamil Nadu, India; sarojinisrnmcchemistry@gmail.com (P.S.); leela232323@gmail.com (K.L.); tkavitha@srnmcollege.ac.in (T.K.); 2 Department of General Pathology, Saveetha Dental College and Hospitals, Saveetha University, Saveetha Institute of Medical and Technical Sciences (SIMATS), Chennai 600077, Tamil Nadu, India; gurushankar01051987@gmail.com; 3 School of Chemical Engineering, Yeungnam University, Gyeongsan 38541, Republic of Korea; 4 Chemical Sciences Department and The Radical Research Centre, Ariel University, Ariel 40700, Israel; 5 Australian Center for Sustainable Development Research and Innovation (ACSDRI), Unit 36/21 South Tce, 9 Adelaide, SA 5000, Australia

**Keywords:** VO/GPB, methyl orange, visible light, stability, photodegradation, antioxidant

## Abstract

In this study, novel V_2_O_5_-decorated garlic peel biochar (VO/GPB) nanocomposites are prepared via the facile hydrothermal technique. As-synthesized VO/GPB is characterized by various spectroscopic and analytical techniques. The surface morphology of the as-prepared samples was predicted by SEM analysis, which shows that the block-like V_2_O_5_ was uniformly decorated on the stone-like GPB surface. The elemental mapping analysis confirms the VO/GPB composite is composed of the following elements: C, O, Na, Mg, Si, P, K, and V, without any other impurities. The photocatalytic activity of the VO/GPB nanocomposite was examined by the degradation of methyl orange (MO) under the irradiation of visible light; 84% degradation efficiency was achieved within 30 min. The reactive oxidative species (ROS) study reveals that hydroxyl and superoxide radicals play an essential role in MO degradation. Moreover, the antioxidant action of the VO/GPB nanocomposite was also investigated. From the results, the VO/GPB composite has higher antioxidant activity compared to ascorbic acid; the scavenging effect increased with increasing concentrations of VO/GPB composite until it reached 40 mg/L, where the scavenging effect was the highest at 93.86%. This study will afford innovative insights into other photocatalytic nanomaterials with effective applications in the field of photocatalytic studies with environmental compensation.

## 1. Introduction

A growing concern at the current time is the release of organic contaminants into the water system. The large scale of untreated wastewater from pharmaceutical industries and agricultural activities, including mineral gases and volatile and semi-volatile organic hydrocarbons, are sources of environmental pollution [1,2,3]. Worldwide, people are regularly exposed to a wide range of toxic pollutants. These kinds of toxic pollutants cause contamination in air, food, and drinking water. Organic pollutants from human and animal waste, as well as industrial byproducts, can affect aquatic creatures and microbes if they are not properly treated before being released into the environment. Azo-containing complexes account for over 70% of the dye family [4,5,6]. Among them, methyl orange (MO) is broadly utilized in foodstuffs, paper, leather industries, and textiles. Because of the improper release of MO in the ecosystem, the contamination of water bodies is highly increased; it must be removed from water bodies, owing to its toxicity [7,8,9]. These kinds of challenging problems increase the need for green, as well as highly effective, methodologies to eliminate toxic pollutants from the environment. Typical analytical methods like ozonation, membrane separation, sonalysis, and coagulation are often inadequate in the handling of vast, new, emerging micro-pollutants [10,11]. 

Photocatalysis is a subject that combines the topics of energy research, nanotechnology, environmental science, photonics, material science, chemical analysis, and so on [12]. Recently, semiconductors have generally been chosen as photocatalysts, since they have a narrow bandgap between the valence and conduction bands. Changing and promoting emergent technology leads to decreased contamination in air and water [13,14,15]. This is the best green approach for furnishing everyone in the world. Semiconductor catalysts are favored in the photocatalytic degradation of wastewater for the following reasons: (i) they are economical; (ii) their non-toxicity; (iii) they show accommodating properties that can be modified by doping, size tailoring, or sensitizers; (iv) they afford the ability for a multi-electron relocate process; and (v) they are able to enlarge their utilization without considerable loss in the photocatalytic application [16,17,18]. TiO_2_ and ZnO are well known to be excellent photocatalysts for the degradation of numerous environmental contaminants, owing to their elevated photosensitivity. The most important drawback of TiO_2_ and ZnO is the larger bandgap at 3.2 eV (~388 nm) [19,20]. Because of their large bandgap values, TiO_2_ and ZnO act only in the presence of UV light, with wavelengths shorter than 380 nm.

Recently, various metal oxide nanomaterials have been used for environmental applications [21,22,23]. Among them, V_2_O_5_-based nanomaterials have been synthesized and utilized in a miscellany of applications. Moreover, V_2_O_5_ is an effective n-type semiconductor photocatalyst for the catalytic degradation of hazardous pollutants, due to its harmless nature, morphology, and simulated sunlight absorption [24,25,26]. Due to its quick electron–hole recombination and limited stability, bare V_2_O_5_ is inadequate to meet the realistic necessities in photocatalytic applications [27]. The inclusion or doping of various metals, semiconductors, or other suitable elements to improve the catalytic stability of V_2_O_5_ is an excellent alternative [28,29]. 

Production of carbon-based nanomaterials, i.e., biochar nanocomposites, has resulted from the combination of biochar technology and nanobiotechnology. Improved physical, chemical, and surface qualities can be found in nanosized biochar material. Among its many uses, it can be a soil amendment, an effluent purification aid, a bioremediation substrate, a plant disease surveillance tool, and a support substance for inhibiting enzymes. Its low price, long lifespan, and low impact on the surroundings make it a strong contender to replace more established methods. The retention of biochar is another way it helps with climate change mitigation. Because of its mobility in soil and superior absorption capacity, biochar could be a viable alternative for disposing of waste. Biochar is a thermally processed biomaterial that is made from plant waste through pyrolysis and serves as a vital supporting material. It is a charcoal-like material with a specific morphology and a large surface area that provides better catalytic activity against organic pollutants [30,31,32]. For these and other reasons, biochar is increasingly being included in nanocomposites [17,33,34,35,36,37] for applications like wastewater treatment, energy generation, and the adsorption of organic dyes and other pollutants. Its porous structure, increased number of exterior functional groups, and better surface area-to-volume ratio make it a successful material for enzymes [38]. Biochar is a cost-effective and environmentally sustainable material that exhibits exceptional efficiency and can be easily produced. Wastewater treatment has garnered heightened attention. Biochar possesses several characteristics that render it a good material for pollution treatment. These include its high surface area, high porosity, presence of sufficient functional surface groups, exceptional ion exchange ability, and remarkable stability. Furthermore, the synergistic interaction between biochar and various metals or metal oxides has the potential to augment the adsorption capability, amplify the absorption of visible light, facilitate the separation of photogenerated electrons and holes, diminish the band gap, and, ultimately, enhance the photocatalytic efficacy of catalysts based on biochar. Extensive research has been conducted on the utilization of biochar and biochar-based products for the remediation of environmental contaminants [39,40,41]. 

In this work, VO/GPB nanocomposite was prepared by a typical hydrothermal method. The VO/GPB catalyst was analyzed by X-ray Powder Diffraction (XRD), Ultraviolet–Visible Diffuse Reflectance Spectroscopy (UV-DRS), Scanning Electron Microscopy (SEM) with Energy-dispersive X-ray spectroscopy (EDAX), and photoluminescence. Due to their remarkable physical and chemical characteristics, VO/GPB metal oxide nanostructures find widespread application. These include, but are not limited to, biomedical applications, the conversion of carbon dioxide, the photocatalytic destruction of organic pollutants, the purification of pollutants such as heavy metals, the identification of hazardous gases, the surface treatment of fabrics for use in wearable electronic gadgets, and the filtration of heavy metals from the environment. Due to their inexpensiveness, durability, effectiveness, and limited influence on the atmosphere, these types of metal oxides are widely recognized as materials for the remediation of environmental damage. Metal oxides are promising for environmental remediation because of their high catalytic capability when driven by visible light. Metal oxides’ electrical structure, light-absorbing properties, charge-transport mechanisms, and enhanced lifetimes all work together to make them good photocatalysts. This research looks at the recent trends and possible future possibilities for using metal oxides as photocatalysts in the fields of energy and the environment. In addition, it provides a thorough evaluation of the state of the industry, the challenges it faces, and the potential for expansion. Taking these factors into account, we developed VO/GPB nanocomposites that are inexpensive, extremely effective, lightweight, and kind to the environment. In just 30 min, the photocatalytic degradation efficiency of MO dye was increased to almost 84% under visible light irradiation. In photocatalytic applications, the VO/GPB nanocomposite has shown remarkable degrading efficacy and high stability. This is the first publication to our knowledge to discuss the use of VO/GPB nanocomposites as a photocatalyst for the degradation of MO dye, and the results show significantly higher degradation efficiency values than those shown in previous studies. Additionally, the antioxidant activity of the VO/GPB nanocomposite was studied to predict its ability to scavenge the hydroxyl radical. Therefore, it gives researchers in this field new information to consider. 

## 2. Materials and Methods

Analytical grade ammonium metavanadate (NH_4_VO_3_) ≥ 99.0%, cetyl trimethyl ammonium bromide (CTAB) ≥ 98%, sodium hydroxide (NaOH) ≥ 98%, methyl orange (MO) 85%, benzoquinone (BQ) ≥ 98%, triethanolamine (TEA) ≥ 99%, and isopropyl alcohol (IPA) ≥ 99.7% were purchased from Sigma-Aldrich (St. Louis, MO, USA). All other chemicals and reagents were analytical grade and used without further purification. All the required solutions were prepared with distilled water.

### 2.1. Preparation of Garlic Peel Biochar (GPB)

The garlic was collected in a single batch from a local store in Krishnankoil, Tamil Nadu, India. After collection, the garlic was peeled, cleaned, and dried overnight at 110 °C. The dried peels were pulverized to obtain a fine powder. The powdered garlic peel was subjected to calcination at 580 °C for 6 h. As prepared, garlic peel biochar was named GPB.

### 2.2. Preparation of V_2_O_5_/Garlic Peel Biochar (VO/GPB) Composite

**Figure 1:**
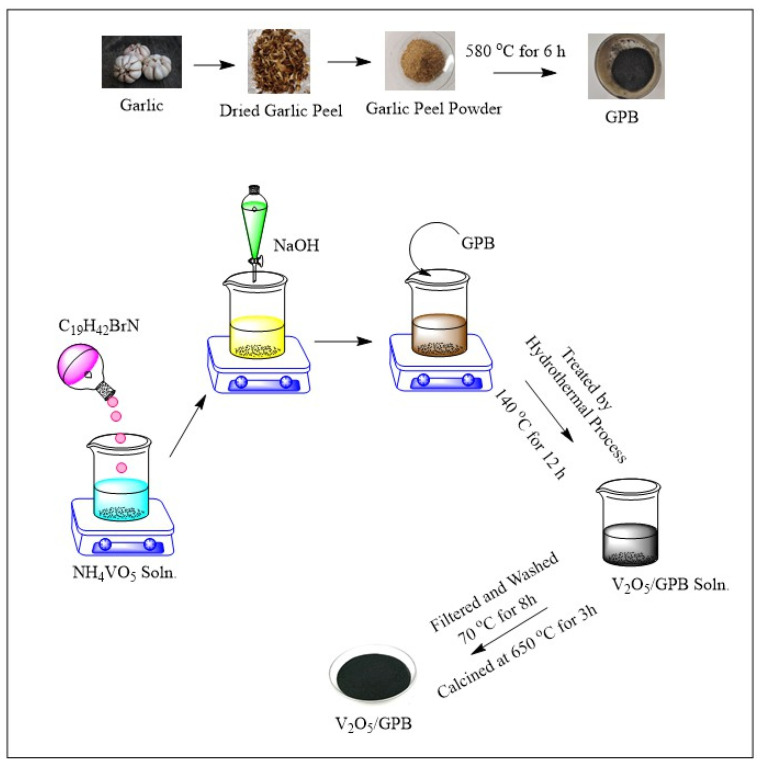
Schematic representation of preparation of VO/GPB composite.

In a simple hydrothermal method, 0.1 mol of NH_4_VO_3_ and 0.1 mol of C_19_H_42_BrN were completely dissolved in 100 mL of distilled water with continuous stirring for 1 h. NaOH was used to adjust the pH of the solution to 10. Consequently, 0.5 g of GPB was mixed with the above solution. This solution was allowed to undergo ultra-sonification for 30 min. Then, after stirring, the mixture was placed in a Teflon-lined autoclave and heated to 140 °C for 12 h. Water and ethanol were used to wash and centrifuge the final product. Finally, the VO/GPB composite was calcined at 650 °C for 3 h. Figure 1 shows the schematic representation of the preparation of the VO/GPB composite.

### 2.3. Characterization Technique

The XRD patterns of VO/GPB samples were analyzed using a Bruker-D8 Advance ECO X-ray diffractometer (Shimadzu Corporation, Kyoto, Japan). The IR Tracer-100 FT-IR spectrophotometer was used to examine the Fourier-transform infrared spectroscopy (FT-IR) spectrum (Jasco, Tokyo, Japan). The morphology of the VO/GPB samples was recorded by EVO18-CARL ZEISS (Oberkochen, Germany) Scanning Electron Microscopy. The UV–Visible-Diffuse Reflectance Spectrum (UV-DRS) was predicted by Shimadzu UV-2600 (Shimadzu Corporation, Kyoto, Japan), in which BaSO_4_ was used as reference material. The photoluminescence was analyzed by RF-6000 (Shimadzu Corporation, Kyoto, Japan). The UV-1800 Shimadzu UV–visible spectra were utilized to analyze the concentration of the photodegradation solution. Origin 8.5 software was used to draw a plot in this work.

### 2.4. Photocatalytic Experiments

The photocatalytic action of VO/GPB was estimated by MO degradation under the illumination of visible light equipped with a tungsten incandescent lamp (500 W). Consequently, 40 mg/L of the VO/GPB nanocomposite was dissolved in 100 mL of distilled water with a 30 mg/L concentration of MO. Then, the solution was stirred for 30 min to maintain adsorption–desorption equilibrium. The above solution was placed in a reaction chamber. At a specific interval, an aliquot sample was collected, and the catalyst was excreted through an ultracentrifuge. The supernatant solutions were analyzed by a UV–Visible spectrophotometer to identify the concentration of the solution. At last, the VO/GPB nanocomposite was collected for the repeatability test. In addition, the photocatalytic degradation efficiency of VO/GPB was calculated by the following equation:

Photodegradation efficiency (%) = (C_0_ − C/C_0_) × 100

where C_0_ and C correspond to the initial and final concentration of MO dye before and after irradiation. 

### 2.5. Antioxidant Activity by DPPH Assay

The antioxidant behavior of the VO/GPB nanocomposite was examined by the 1-1-diphenyl-2-picryl-hydrazyl (DPPH) free radical scavenging activity. For this, 0.2 μM of 2,2-diphenyl-1-picrylhydrazyl (DPPH) solution was prepared with methanol. Then, 3 mL of VO/GPB nanocomposite (20–40 mg/L) was added to the methanolic solution of DPPH with continuous stirring for 30 min under dark conditions. The absorbance of the above mixture was calculated at 517 nm using a UV-1800 Shimadzu UV/Vis spectrophotometer. The percentage of free radical scavenging activity was measured by the following calculation:

Scavenging activity (%) = {(A_0_ − A_1_)/A_0_} × 100 (1)

where A_0_ and A_1_ are the absorbances of the control and sample, respectively.

## 3. Results and Discussion

### 3.1. XRD Analysis

**Figure 2:**
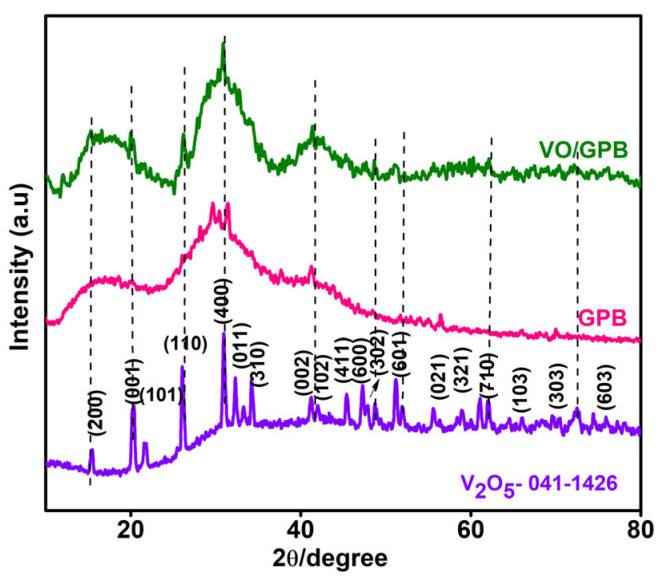
XRD patterns of V_2_O_5_, GPB, and VO/GPB nanoparticles.

The crystalline structure and phase purity of synthesized V_2_O_5_, GPB, and VO/GPB composite were examined using Powder X-ray Diffraction (PXRD) analysis, as depicted in Figure 2. The Powder-XRD (PXRD) patterns of pristine V_2_O_5_ were perfectly matched with standard JCPDS Card No. 41-1426 [42]. It showed peaks at 2θ values of 15.37, 20.26, 21.95, 26.34, 30.72, 32.41, 34.45, 41.18, 45.56, 47.43, 51.13, 55.85, 58.90, 60.77, 62.10, 65.97, 72.05, and 74.43°, corresponding to (200), (001), (101), (110), (400), (011), (310), (002), (411), (600), (302), (601), (021), (321), (710), (103), (303), and (503), confirming the crystalline nature of V_2_O_5_. The VO/GPB composite had major PXRD peaks for V_2_O_5_ and GPB. Moreover, the orientation of the peak was shifted to a higher intensity due to doping with GPB. This proves the successful formation of VO/GPB nanocomposite. There was no other peak from impurities, showing the purity of the prepared samples.

### 3.2. Morphological Analysis

**Figure 3:**
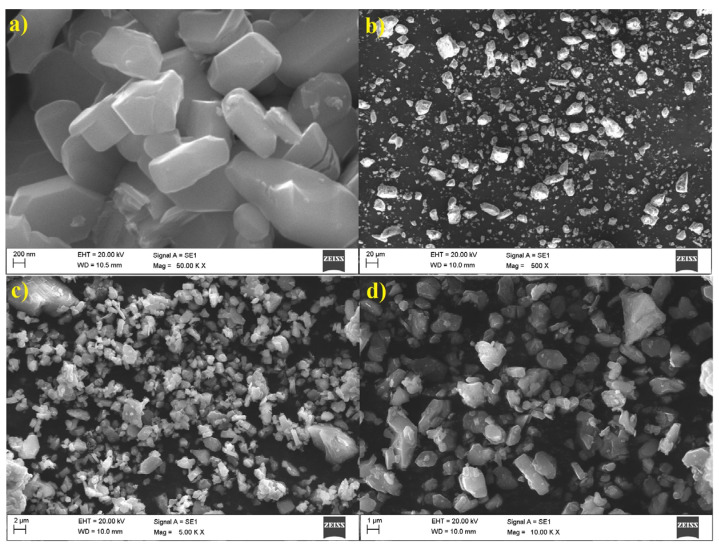
SEM image of as-prepared (**a**) V_2_O_5_ nanoparticles captured at ×50,000 (scale bar 200 nm), (**b**) GPB captured at ×500 (scale bar 20 μm) and (**c**,**d**) VO/GPB nanocomposite captured at ×5000 (scale bar 2 μm) and ×10,000 (scale bar 1 μm) at different magnifications.

The morphological analysis of V_2_O_5_, GPB, and VO/GPB composite was performed using Scanning Electron Microscopy (SEM) analysis. As shown in Figure 3a, the SEM revealed that the bare V_2_O_5_ nanomaterials exhibited a block-like structure as surface morphology. This demonstrates that they were nanometer-sized, and Figure 3b reveals the stone-like GPB morphology structure, which was well agglomerated. 

**Figure 4:**
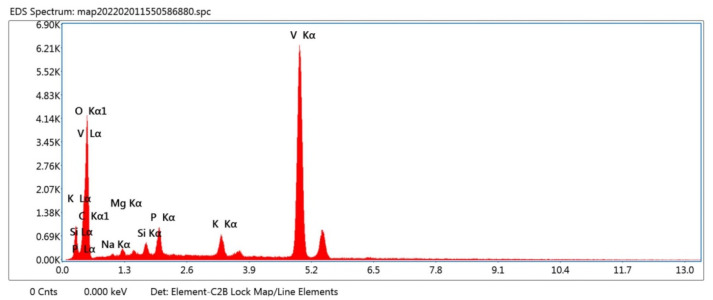
EDAX spectrum of as-prepared VO/GPB composite.

**Figure 5:**
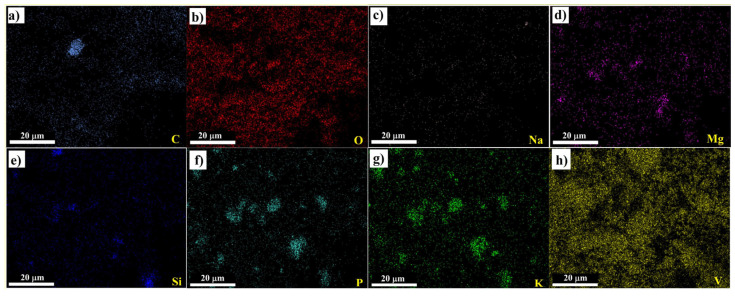
Elemental mapping images with a magnification of ×500 (scale bar 20 μm) of VO/GPB composite. The color mapping analysis of various components (**a**) C, (**b**) O, (**c**) Na, (**d**) Mg, (**e**) Si, (**f**) P, (**g**) K and (**h**) V.

Figure 3c,d demonstrates the different magnifications of the VO/GPB composite: the V_2_O_5_ was finely dispersed in GPB stones in the composite structure. The size of the VO/GPB composite ranged from 200 nm to 1 μm. Furthermore, EDAX (Figure 4) analysis was used to estimate the total weight of the different elemental compositions presents in the VO/GPB nanocomposite. The results of EDAX and elemental mapping analysis (Figure 5a–h) confirmed that the VO/GPB composite was composed of C, O, Na, Mg, Si, P, K, and V, without any other impurities. Additionally, the weight percentages of the elements present in the composite were 6.8, 23.4, 0.9, 1.4, 1.4, 3.4, 3.2, and 59.5 wt.%, respectively.

### 3.3. Optical Properties

**Figure 6:**
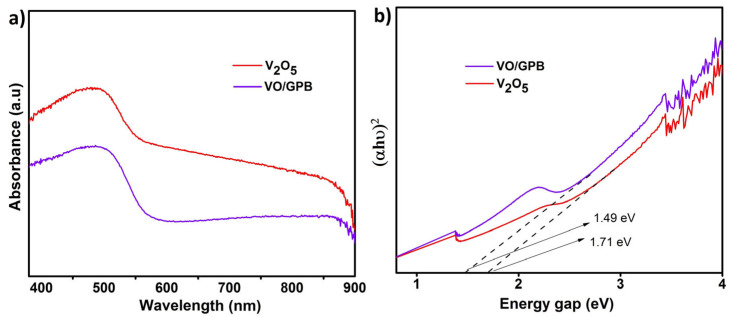
Optical properties of samples: (**a**) UV–Vis diffused reflectance spectra of as-prepared V_2_O_5_ and VO/GPB samples, and (**b**) the bandgap energy values of V_2_O_5_ and VO/GPB nanocomposite.

The UV-DRS analysis was used to estimate the bandgap value for the V_2_O_5_ nano-blocks, GPB grits, and VO/GPB composite, and also to predict the irradiation source for the photocatalytic degradation. A bandgap value plays an important role in this photocatalytic process [43]. Based on this, the catalysts absorb light energy and initiate the recombination of electron (e^−^)–hole (h^+^) pairs [44,45]. From Figure 6a, the VO/GPB composite had an absorption value of nearly 500 nm; this high absorption wavelength proved that the photocatalytic degradation should perform well under visible light source irradiation. As shown in Figure 6b, Tauc’s plots equation was utilized to identify the band energy values of the V_2_O_5_ nanoblocks and VO/GPB composite, which were 1.71 and 1.49 eV, respectively. The bandgap values of VO/GPB were reduced by combining them with GPB biochar in the V_2_O_5_ sample. The visible light photocatalytic activity of the nanocomposite is enhanced by the incorporation of biochar due to the reduction in the overall optical bandgap and the minimization of the electron–hole (e–h) recombination rate. And finally, it prevents photo-induced electron–hole pairs from recombining and speeds up the electron transfer process from photocatalyst particles to reactive sites. While V_2_O_5_ has a band gap energy of about 1.71 eV, for VO/GPB, the value was only reduced to the 1.49 eV range when biochar was also included.

### 3.4. Photoluminescence Studies

**Figure 7:**
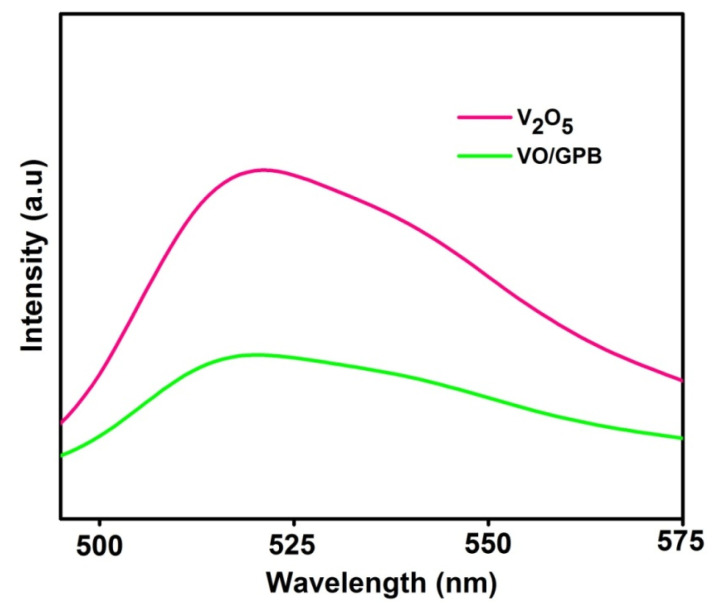
The Photoluminescence spectra of the as-prepared V_2_O_5_ and VO/GPB samples.

The Photoluminescence (PL) spectrum of V_2_O_5_ nanoblocks and VO/GPB composite is displayed in Figure 7. PL analysis is used to estimate the charge separation between photo-generated e^−^ − h^+^ pairs. The V_2_O_5_ nanoblocks show an excitation peak at 520 nm, owing to the recombination of the e^−^ − h^+^ pair. But the VO/GPB composite exhibited a lower intensity peak than the V_2_O_5_ nanoblocks due to the lower recombination of charge carriers. This lesser charge separation of e^−^ − h^+^ pairs improves the lifetime of the charge carriers and increases the catalytic efficiency of the VO/GPB composite. 

### 3.5. Estimation of the Photodegradation Process

#### 3.5.1. The Effectiveness of the Diverse Catalyst

**Figure 8:**
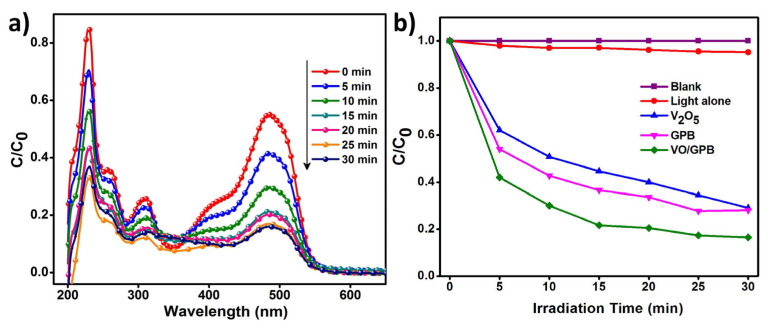
Photodegradation of MO solution under different conditions: (**a**) Absorption spectrum for photodegradation of aqueous MO solution under visible light irradiation in the presence of 40 mg VO/GPB catalyst and (**b**) Photocatalytic degradation of MO dye of different samples under visible light irradiation.

The catalytic activity of the V_2_O_5_ nanoblocks, GPB grits, and VO/GPB composite were examined by the degradation of methyl orange (MO—30 mg/L) by irradiation with visible light. The photodegradation absorption spectra of MO are shown in Figure 8a. In the degradation process, the electron–hole pairs are generated by the illumination of light; after 30 min of irradiation, the concentration of MO slowly diminished to almost zero. The VO/GPB composite showed degradation efficiency of up to 84%. Based on this process, the effects of V_2_O_5_ nanoblocks, GPB grits, and VO/GPB composite catalytic materials were predicted. As shown in Figure 8b, the VO/GPB composite possesses a superior catalytic efficiency than the bare V_2_O_5_ nanoblocks and GPB grits, owing to the lower energy gap value and considerable light absorption in the visible region. Moreover, the optimal parameters like catalyst loading and dye concentration for the photodegradation process were considered to organize the optimal conditions for the degradation of MO.

#### 3.5.2. The Effectiveness of Catalyst Dosage

**Figure 9:**
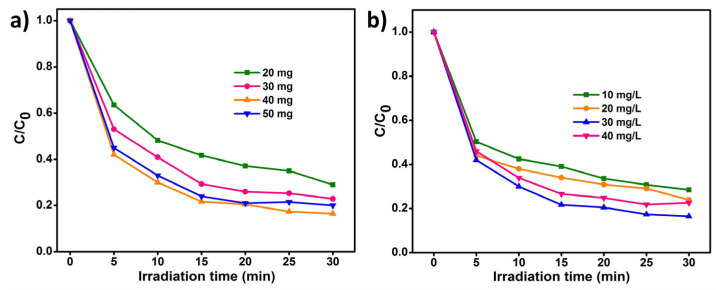
Photodegradation of MO dye at different conditions: (**a**) Different loading dosages of VO/GPB composite and (**b**) Different concentrations of MO dye in aqueous solution.

The MO degradation (40 mg/L) with V_2_O_5_ nanoblocks, GPB grits, and VO/GPB composite was carried out to find the absorption wavelength under identical conditions. Figure 9a demonstrates the photodegradation of MO when altering the catalyst dose from 20 to 50 mg. Among these values, 40 mg of catalyst was adequate for superior degradation. Additionally, increasing the catalyst dose from 40 to 50 mg decreased the degradation efficacy due to the permeation of light sources into the reaction solution being restricted by the lowest amount of photosynthetic charge carrier production. Thus, 40 mg of catalyst was fixed as the optimum dosage for further reactions. 

#### 3.5.3. The Effectiveness of Pollutant Concentration

The role of pollutant concentration is most important for an effective photocatalytic process. Here, the concentration of MO was altered from 10 to 40 mg/L. From Figure 9b, the efficiency of the catalytic degradation reaction was suppressed, owing to the opacity of the higher concentration of pollutant (30 mg/L of MO). The higher concentration of MO reduces the penetration of the light source into the solution, which encourages a light-scattering effect. Based on this, the lower concentration of pollutants (30 mg/L of MO) shows superior catalytic efficiency compared to higher concentrations.

#### 3.5.4. Effect of pH

**Figure 10:**
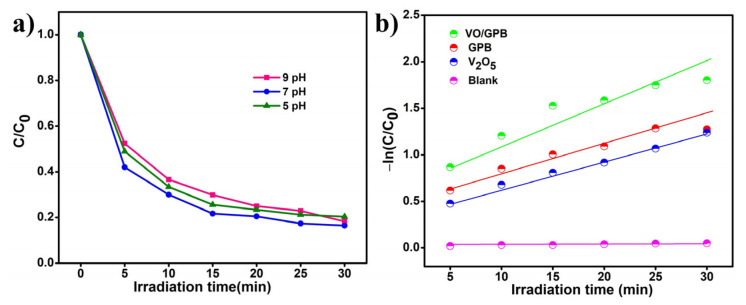
Photodegradation of MO dye with the effects of (**a**) various pH conditions and (**b**) the corresponding first-order kinetics plots for different samples.

Figure 10a shows the outcome of altering the pH of the reaction from 3 to 7 by the use of HCl (acidic medium) and NaOH (basic medium). The VO/GPB composite that reveals better catalytic activity at pH = 3 may be due to the highly protonated photocatalyst surface. The catalytic efficiency of the VO/GPB composite gradually decreased at a high pH value because the surface of the photocatalyst became more negative. As a result, the pH = 3 medium was most favorable for the photodegradation reaction of MO. 

#### 3.5.5. Kinetic Studies

The kinetic studies of V_2_O_5_ nanoblocks, GPB grits, and VO/GPB composite were performed to find the rate constant value of the degradation process, as shown in Figure 10b. The plot of the kinetic studies was drawn between time and ln(C_0_/C) value. V_2_O_5_ nanoblocks, GPB grits, and VO/GPB composite showed rate constant values of 0.0367, 0.0385, and 0.0539 min^−1^, and the R^2^ values were 0.9168, 0.8202, and 0.7956, respectively. The VO/GPB composite shows higher rate constant values than the others; this is due to the photoelectric separation capacity before the renovation of pollutant irradiation.

#### 3.5.6. ROS Study

**Figure 11:**
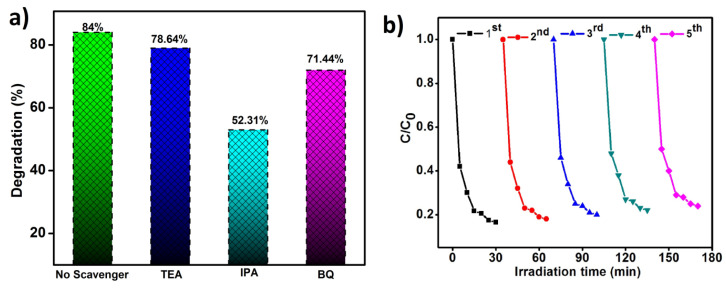
ROS study and the recycle ability analysis of VO/GPB samples: (**a**) Photodegradation of MO dye in the presence of various scavengers, (**b**) Repeatability experiments for the different reactions with the as-prepared VO/GPB nanocomposite under visible light irradiation.

The Reactive Oxidative Species (ROS) study was used to forecast the effect of scavengers in the photodegradation process. Different scavengers like TEOA, IPA, and BQ were used as quenchers for h^+^, ^•^O_2_^−^, and ^•^OH, respectively, to examine the main reactive species in the degradation process [46,47]. As shown in Figure 11a, upon the irradiation by visible light, the degradation of MO was decreased due to the addition of IPA and BQ as quenchers for ^•^OH and ^•^O_2_^−^, respectively. But a very tiny range of changes was observed, while the addition of TEOA acted as a quencher for h^+^. These results prove that the ^•^OH and ^•^O_2_^−^ radicals play a major role in the degradation of MO solution under the illumination of visible light.

#### 3.5.7. Plausible Mechanism of the Photodegradation

Based on the above set of results, the mechanism of the MO degradation was predicted as follows:

VO/GPB + hʋ → VO/GPB (h^+^_VB_)/VO/GPB (e^−^_CB_) (2)

VO (e^−^ + h^+^)/GPB (e^−^ + h^+^) → VO (e^−^ + e^−^)/GPB (h^+^ + h^+^) (3)

(e^−^_CB_) + O_2_ → ^•^O_2_^−^ (4)

(e^−^_CB_) + 2H^+^ + ^•^O_2_^−^ → ^•^OH + OH^−^ (5)

(h^+^_VB_) + OH^−^ + H_2_O → ^.^OH + H^+^ (6)

^•^O_2_^−^ + ^•^OH + h^+^ + MO → CO_2_ + H_2_O + Mineral acids (7)

Upon the irradiation by visible light, the VO/GPB composite becomes excited and generates electrons in the conduction band (CB), at the same time leaving holes (h_vb_^+^) in the valence band, as depicted in the Equations (2) and (3). From Equation (4), the superoxide radical anion (^•^O_2_^−^) is generated by the reaction between the electrons (e^−^) and an atmospheric oxygen molecule. As shown in Equation (5), superoxide radical anions react with an excess of electrons (e^−^) and holes (h_vb_^+^) to form hydroxyl radicals (^•^OH). After that, the h_vb_^+^ reacts with ^•^OH and water molecules to form ^•^OH and H^+^ ions (Equation (6)). Finally, the superoxide radical anion (^•^O_2_^−^) reacts with ^•^OH and h^+^ to convert the MO pollutant into non-toxic CO_2_, H_2_O, and mineral acids, as depicted in Equation (7).

#### 3.5.8. Reusability Test

After the successful degradation reaction, the catalyst was collected to predict its stability and reusable capacity. Figure 11b attributes the effect of the reusability test to MO degradation within 30 min. The VO/GPB composite produces better catalytic efficiency up to the fifth cycle of degradation. If the test was raised after the fifth cycle, the efficacy of the catalyst was diminished. The efficiency of the VO/GPB composite for MO degradation was 83.86%, 81.21%, 79.91%, 77.05%, and 75.37% for five cyclical runs. This result confirms the mechanical stability and reusability properties of VO/GPB composite. Additionally, Table 1 compares VO/GPB with other catalysts for the degradation of different contaminants that have been reported. With a catalyst dose of 0.04 g/L, this also demonstrated improved performance on the 84% degradation of MO in 30 min. Alternatively, other catalysts need at least 90 min to degrade the pollutants. 

**Figure 12:**
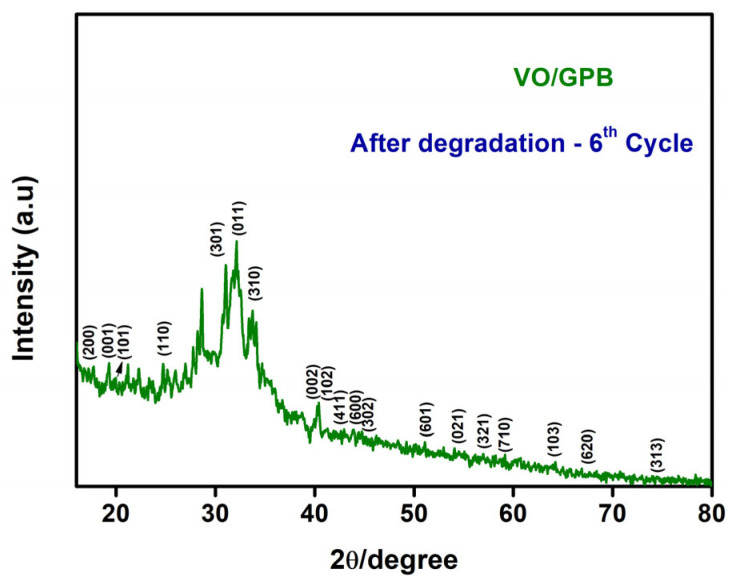
XRD analysis of VO/GPB nanocomposite after 6th-cycle degradation of MO dye.

In Figure 12, we see the XRD analysis of the VO/GPB nanocomposite following the sixth cycle of MO dye degradation. The durability and recyclability of the produced nanocomposites were demonstrated by the fact that the VO/GPB photocatalyst showed only a slight drop in photocatalytic efficiency rather than a large loss after six cycles, with increased photocatalytic efficacy attained. In addition, XRD tests performed after six cycles provide conclusive evidence of the purity of the synthesized nanocomposite.

### 3.6. Antioxidant Activity

**Figure 13:**
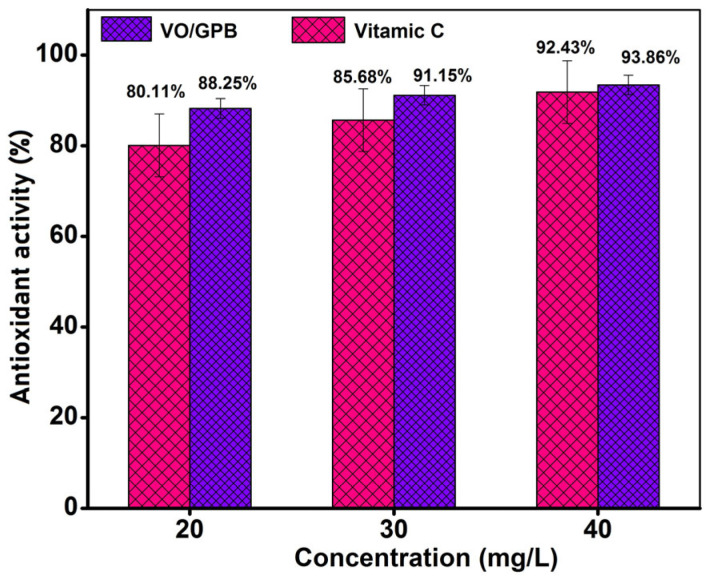
Percentage of antioxidant activity of VO/GPB composite using Vitamin C as standard.

According to the literature, most metal oxides have antioxidant activity and, thus, they are emerging as effective antioxidants and therapeutic reagents. In general, the hydroxyl free radical is accountable for tissue damage and is associated with inflammation; hence, the removal of this free radical is acknowledged as the main goal of antioxidant treatment [55,56,57,58,59]. The DPPH assay experiment was applied to examine the antioxidant activity of synthesized VO/GPB composite in the varied concentration range of 20–40 mg/L using ascorbic acid (Vitamin C) as a standard drug in this work. DPPH’s radical-scavenging ability is superior, and it creates a stable free radical that can accept an electron or hydrogen [60,61]. The free radical can then be converted into a diamagnetic compound that is stable. The reaction mixture was incubated at room temperature for 30 min, and absorbance was measured at 517 nm, which showed a significant absorption band. As the electron becomes coupled, the absorption reduces, as the number of electrons taken up increases [62,63]. This type of change in absorption has long been used to assess a VO/GPB composite capacity to function as an antioxidant. These findings show that the VO/GPB composite has higher antioxidant activity compared to ascorbic acid; the scavenging effect increased with increasing concentrations of VO/GPB composite until it reached 40 mg/L, where the scavenging effect was the highest, at 93.86%, which confirms almost identical free radical inhibitory action. Figure 13 depicts the antioxidant efficacy of the VO/GPB composite. Table 2 shows the comparison report for the antioxidant activity percentage by Vitamin C and VO/GPB. From the results, VO/GPB nanocomposite has higher antioxidant activity values than the standard Vitamin C at various concentrations of VO/GPB samples.

## 4. Conclusions

In summary, the VO/GPB composite has been fruitfully fabricated via an easy hydrothermal path. The morphology and crystallinity of V_2_O_5_, GPB, and VO/GPB composite were established and characterized by PXRD, SEM with EDAX, UV-DRS, and PL analysis. From the UV-DRS investigation, the bandgap values of prepared samples were predicted, and the VO/GPB composite showed a suitable band energy value compared to bare V_2_O_5_. Providentially, the VO/GPB composite exhibited superior photocatalytic effectiveness in the visible light region. It showed tremendous charge carrier separation and catalytic activity towards the degradation of MO, with 84% degradation efficacy within 30 min. The VO/GPB composite demonstrated elevated stability and repeatability over five repeated runs. This study proposed a new point of view on the growth of photodegradation by utilizing a VO/GPB composite for ecological applications. In addition, the VO/GPB composite had higher antioxidant activity compared to ascorbic acid at 40 mg/L, where the radical-scavenging effect was the highest, at 93.86%. Due to their low cost, stability, efficiency, and limited impact on the environment, bio-inspired nanometal oxides have become popular as materials for environmental remediation. Metal oxides are promising for environmental cleanup because of their high catalytic capability when driven by visible light. Metal oxides have been investigated for their potential as photocatalysts in a variety of energy and environmental applications, with a focus on recent advances and future directions in this field. Hydrogen production by water splitting is crucial, as is the growth of renewable energy in general.

## Figures and Tables

**Table 1:** Comparison of degradation performance using VO/GPB with various catalysts.

S. No	Catalyst	Weight of Catalyst (g/L)	Organic Pollutant	Irradiation Light	% of Degradation	Time (min)	Source
1	TiO_2_–bamboo	0.05	MB	UV light	90	120	[48]
2	BiOX (X = Cl or Br)–biochar	0.05	MO	Visible light	82	150	[49]
3	g-C_3_N_4_–biochar	0.45	MB	LED light	91	240	[50]
4	Nano-β-FeOOH/Fe_3_O_4_/Biochar	0.1	MO	Xenon lamp	98	90	[51]
5	TiO_2_/Fe/Fe_3_C/biochar	1.00	MB	UV light	89.2	300	[52]
6	Fe_2_O_3_/TiO_2_/biochar	2.00	MB Rh BMO	Visible light	75 60 40	60	[53]
7	Zn/TiO_2_/biochar	1.25	Sulfamethoxazole	Visible light	80.0	180	[54]
8	VO/GPB	0.04	MO	Visible light	84	30	This work

**Table 2:** The antioxidants efficiency of VO/GPB composite using Vitamin C as standard.

Concentration (mg/L)	Vitamin C (%)	VO/GPB (%)
20	80.11	88.25
30	85.68	91.15
40	92.43	93.86

## Data Availability

Not applicable.
